# Effect of Resistance Training and Fish Protein Intake on Motor Unit Firing Pattern and Motor Function of Elderly

**DOI:** 10.3389/fphys.2018.01733

**Published:** 2018-12-04

**Authors:** Kohei Watanabe, Aleš Holobar, Yukiko Mita, Motoki Kouzaki, Madoka Ogawa, Hiroshi Akima, Toshio Moritani

**Affiliations:** ^1^Laboratory of Neuromuscular Biomechanics, School of International Liberal Studies, Chukyo University, Nagoya, Japan; ^2^Faculty of Electrical Engineering and Computer Science, University of Maribor, Maribor, Slovenia; ^3^Department of Human Nutrition, School of Life Studies, Sugiyama Jogakuen University, Nagoya, Japan; ^4^Laboratory of Neurophysiology, Graduate School of Human and Environmental Studies, Kyoto University, Kyoto, Japan; ^5^Research Center of Health, Physical Fitness and Sports, Nagoya University, Nagoya, Japan; ^6^Graduate School of Education and Human Development, Nagoya University, Nagoya, Japan; ^7^Faculty of Sociology, Kyoto Sangyo University, Kyoto, Japan

**Keywords:** aging, nutritional supplementation, multichannel surface electromyography, motor unit decomposition, countermeasures to aging

## Abstract

We investigated the effect of resistance training and fish protein intake on the motor unit firing pattern and motor function in elderly. Fifty healthy elderly males and females (69.2 ± 4.7 years) underwent 6 weeks of intervention. We applied the leg-press exercise as resistance training and fish protein including Alaska pollack protein (APP) as nutritional supplementation. Subjects were divided into four groups: fish protein intake without resistance training (APP-CN, *n* = 13), placebo intake without resistance training (PLA-CN, *n* = 12), fish protein intake with resistance training (APP-RT, *n* = 12), and placebo intake with resistance training (PLA-RT, *n* = 13). Motor unit firing rates were calculated from multi-channel surface electromyography by the Convolution Kernel. For the chair-stand test, while significant increases were observed at 6 weeks compared with 0 week in all groups (*p* < 0.05), significant increases from 0 to 3 weeks and 6 weeks were observed in APP-RT (18.2 ± 1.9 at 0 week to 19.8 ± 2.2 at 3 weeks and 21.2 ± 1.9 at 6 weeks) (*p* < 0.05). Increase and/or decrease in the motor unit firing rate were mainly noted within motor units with a low-recruitment threshold in APP-RT and PLA-RT at 3 and 6 weeks (12.3 pps at 0 week to 13.6 pps at 3 weeks and 12.1 pps at 6 weeks for APP-RT and 12.9 pps at 0 week to 13.9 pps at 3 weeks and 14.1 pps at 6 weeks for PLA-RT at 50% of MVC) (*p* < 0.05), but not in APP-CN or PLA-CN (*p* > 0.05). Time courses of changes in the results of the chair-stand test and motor unit firing rate were different between APP-RT and PLA-RT. These findings suggest that, in the elderly, the effect of resistance training on the motor unit firing rate is observed in motor units with a low-recruitment threshold, and additional fish protein intake modifies these adaptations in motor unit firing patterns and the motor function following resistance training.

## Introduction

The effect of exercise and nutrition interventions on muscle mass and function in the elderly have been investigated in numerous studies, and they have been accepted as essential to prevent age-related muscle atrophy and dysfunction (Dickinson et al., 2013). However, some studies pointed out that reductions in the muscle strength of the elderly cannot be explained solely by a loss in muscle volume (Delmonico et al., 2009), and the age-related dysfunctions in central nervous system such as decreases in motor unit firing rate and recruitment induced by decrease in supraspinal drive and/or spinal excitability, would also be closely associated with any age-related decrement in muscle strength (Clark and Manini, 2008; Manini and Clark, 2012). In fact, while several studies reported age-related declines in muscle volume in adult humans (Young et al., 1984, 1985; Deschenes, 2004; Abe et al., 2011), some previous studies reported that motor unit firing properties, which are one of indicators of age-related dysfunctions in the central nervous system, are markedly affected by aging (Soderberg et al., 1991; Kamen et al., 1995; Erim et al., 1999; Roos et al., 1999; Watanabe et al., 2016). Also, Moritani and deVries (1980) demonstrated the greater contribution of neural factor to an increase in muscle strength following resistance training compared with muscle hypertrophy in elderly men (Moritani and deVries, 1980). Our previous study identified a positive correlation between motor unit firing pattern of the vastus lateralis (VL) muscle and MVC force of the quadriceps femoris muscles in the elderly (Watanabe et al., 2016). This suggests that, in addition to the muscle volume or peripheral muscle morphology, the central nervous system plays an important role in physiological mechanisms leading to the age-related decline in muscle strength and its countermeasures. However, quantification of the detailed functioning of the central nervous system is more difficult than that of the muscle mass due to methodological limitation.

Motor unit activation properties are used to assess and quantify the role of the central nervous system in human movement. Intramuscular EMG with wire or needle electrodes was applied in previous studies to detect motor unit action potentials (Merletti and Farina, 2009). Although this technique is available for clinical research and it can provide important information, it is less suitable for application to the elderly and repeated measurements, such as conducted in an intervention study. Recent studies attempted to develop new methodologies to non-invasively detect motor units using the multi-channel surface EMG technique (Holobar and Zazula, 2004; Merletti et al., 2008; Farina et al., 2010). This novel technique can be applied for the needle intolerant subjects and in experimental protocols where the intramuscular EMG technique is not desirable or possible, such as an intervention study involving the elderly. Also, the multi-channel surface EMG technique can detect firing patterns from larger population of motor units when compared with the intramuscular technique. Moreover, the multi-channel surface EMG technique facilitates the robust assessment of motor unit firing rates along with their recruitment thresholds (Holobar et al., 2009). Our previous study showed that a decrease in the firing rate of the elderly, which was reported in other studies (Soderberg et al., 1991; Kamen et al., 1995; Erim et al., 1999; Roos et al., 1999), mainly occurs in motor units recruited at a low force level, i.e., ∼20% of MVC (Watanabe et al., 2016). Since it is known that firing rate behaviors are inconsistent among motor units with different recruitment thresholds (De Luca et al., 1982; Erim et al., 1996; Oya et al., 2009; De Luca and Hostage, 2010), the calculation of firing rates for individual motor units with different recruitment thresholds would be necessary to clarify the detailed motor unit firing pattern.

Some evidences that the effect of exercise on the muscle strength and volume in aged individuals can be enhanced by nutritional supplementation has been reported. Most studies focused on protein supplementation to improve muscle synthetic processes in elderly (Dickinson et al., 2013). Also, the effectiveness of some other nutritional supplements such as omega-3 fatty acids, creatine, and β-hydroxy β-methylbutyrate, to promote muscle functions were reported (Fragala et al., 2015). Recently, attention has been paid to traditional natural foods to improve age-related muscle functions (Abe et al., 2017; Abe and Misaka, 2018). For example, the intake of fish protein, i.e., APP, which Japan and other countries have traditionally consumed in daily meals, induced more marked muscle hypertrophy comparing with casein in rat muscle (Mizushige et al., 2010; Kawabata et al., 2015). Natural food-based nutritional supplementation would be convenient for long-term use. However, the effect of nutritional supplementation on the motor function with neuromuscular control strategies in aged humans has not been investigated. As stated above, assessment of the central nervous system would be important to understand age-related muscle dysfunction and the effectiveness of countermeasures such as exercise and/or nutritional supplementation.

The aim of this study was to investigate the effect of resistance training and fish protein intake on motor unit firing pattern and motor function in the elderly. We hypothesized that (1) resistance training increases motor unit firing rate as reported in recent study (Vila-Cha et al., 2010) and this adaptation is observed in motor units with low-recruitment thresholds that are mainly affected by aging (Watanabe et al., 2016); (2) fish protein intake promotes an increase in the muscle volume, as reported in animal studies (Mizushige et al., 2010; Kawabata et al., 2015).

## Materials and Methods

### Subjects

Fifty healthy elderly males and females (mean age ± SD: 69.2 ± 4.7 years, range: 61–83 years) participated in this study. The subjects gave written informed consent for the study after receiving a detailed explanation of the purposes, potential benefits, and risks associated with participation. All procedures used in this study were approved by the Research Ethics Committee of Chukyo University (2015-002, 2016-057) and conducted in accordance with the Declaration of Helsinki.

### Experimental Design

Intervention was conducted for 6 weeks. All subjects came to the laboratory three times for measurements: (1) on the 1st day of intervention before it commenced (0 week), (2) at 3 weeks (3 W) and (3) 6 weeks (6 W) after beginning the intervention study. They also came to the laboratory 3 weeks before the 1st day of the intervention and performed all tests that were used in the measurements at 0, 3, and 6 weeks to familiarize themselves with the instrumentation and motor tasks to be used.

All subjects added test meals to normal daily diets during the intervention. We used two types of test meal with (active) and without (placebo) fish protein including 5g/meal of Alaska Pollock Protein (APP). These additional test meals were 180 g (with APP, active) and 150 g (without APP, placebo) of soups with three different tastes such as miso-soup, minestrone, and clam chowder. Number of each taste was controlled among the subjects. Subject ate one test meal per day and ingestion timing was freely chosen from with breakfast, lunch, or dinner by each subject. A randomized, double-blind, placebo-controlled treatment was conducted for test meals. Finally, subjects were divided into four groups: fish protein intake without resistance training (APP-CN), placebo intake without resistance training (PLA-CN), fish protein intake with resistance training (APP-RT), and placebo intake with resistance training (PLA-RT). The age, number of male and/or female subjects, motor functions, and food intake were controlled among the four groups using the measurement data obtained at 3 weeks before the intervention (Table 1).

**Table 1:** Characteristics of the subjects.

	APP-CN	PLA-CN	APP-RT	PLA-RT
*n*	13	12	12	13
n of male	6	6	6	6
Age (years)	69.2 ± 4.1	69.5 ± 2.9	69.8 ± 5.5	68.3 ± 5.9
Height (cm)	156.9 ± 7.1	159.2 ± 6.9	159.9 ± 7.5	161.1 ± 9.1
Body mass (kg)	53.9 ± 8.9	60.3 ± 10.8	56.2 ± 10.8	57.1 ± 8.6
Body fat (%)	26.0 ± 6.6	27.7 ± 5.8	23.4 ± 5.9	25.3 ± 6.0
Muscle mass (kg)	21.5 ± 4.2	23.5 ± 4.7	23.5 ± 5.2	23.3 ± 4.4
Muscle thickness (cm)	3.52 ± 0.63	3.94 ± 0.99	3.84 ± 0.79	3.94 ± 0.52
ST thickness (cm)	0.68 ± 0.30	0.79 ± 0.28	0.78 ± 0.31	0.68 ± 0.25
MVC (N)	340.0 ± 111.3	350.0 ± 87.8	374.6 ± 131.3	356.4 ± 118.1
Chair stand (times)	16.9 ± 3.6	17.1 ± 3.7	18.2 ± 1.9	18.2 ± 3.3
Gait time (sec.)	7.1 ± 0.8	7.5 ± 1.1	7.2 ± 0.9	6.9 ± 1.0


Before and during the intervention, a dietary survey was performed using a food frequency questionnaire (FFQg), which is based on 29 food groups and 10 types of cooking, in order to estimate the energy and nutrient intakes of individual subjects during the past 1–2 months (Takahashi, 2003). We applied this test to investigate the diets of individual subjects during the past 3 weeks. The total energy intake, absolute protein intake, protein, fat, and carbohydrate intakes normalized by the body weight, and changes between the two periods in protein intake were calculated from the questionnaire responses by Japanese national registered dietitians.

For APP-RT and PLA-RT, resistance training was carried out two times a week, with each session consisting of three sets of ten repetitions of bilateral horizontal leg press with a rest interval of 90 ∼ 120 s. At 0 week, 1 repetition maximum (1RM) was measured for the subjects in APP-RT and PLA-RT before first training session. The load intensities of the training were set at 70% of 1RM. Leg-press exercise involved a 3-s cycle of leg extension (concentric) and 3-s cycle of leg flexion (eccentric). Repetition intervals were set by an electrical metronome and the verbal count of the experimenter given at 1-s intervals. This training regimen was designed as standard resistance training (Kraemer and Ratamess, 2004). For the 1st week, 50 and 60% of 1RM were used for the 1st training and 60 and 70% of 1RM were used for the 2nd training to become familiarized with the resistance training. From the 2nd week, 70% of 1RM was used for all sets of training. If 1RM had increased based on measurement at 3 weeks, the load intensity of the training was also increased. All training sessions were performed under the monitoring and guidance of the experimenters.

### Motor Function Tests

The 10-m walking time test, chair-stand test, and MVC during isometric knee extension were employed as motor function tests at 0, 3, and 6 weeks.

Subjects walked along a 14-m straight line at their preferred speed to assess their walking function. The elapsed times until passing 2 and 12 m were recorded using infrared radiation sensors (4 Assist Co., Ltd., Tokyo, Japan) and the time needed to walk 10 m was calculated. Two trials were performed, and the mean time was used for further analysis.

Numbers of repeated standing and sitting movements conducted for 30 s with maximal effort were recorded using a seat sensor (T. K. K. 5805, Takei Scientific Instruments Co., Ltd., Niigata, Japan) as the chair-stand test (Jones et al., 1999). The height of the chair was 40 cm from the floor. During the test, the arms of the subjects were placed on their chest. To confirm that they were sitting on the chair during the test, one leg was raised momentarily during the sitting phase.

For measuring MVC, the subjects were seated comfortably with the right leg fixed in a custom-made dynamometer (Takei Scientific Instruments Co., Ltd., Niigata, Japan) with a force transducer (LU-100KSE; Kyowa Electronic Instruments, Tokyo, Japan) and both hip and knee joint angles flexed at 90° (180° corresponds to full extension). MVC was determined according to our previously reported procedures (Watanabe et al., 2016). Briefly, the subjects were asked to gradually increase their knee extension force from the baseline to maximum in 2–3 s and then sustain it maximally for 2 s. The timing of the task was based on a verbal count given at 1-s intervals, with vigorous encouragement from the investigators when the force began to plateau. The subjects performed at least 2 MVC trials with a ≥2 min rest interval between them. The highest MVC force was used to calculate the MVC torque and target torque for sustained contraction. The knee extension torque was calculated as the product of the knee extension force and distance between the estimated rotation axes for the knee joint in the sagittal plane. The force transducer was located approximately 5 cm proximal to the lateral malleolus on the front of the shank.

### Multi-Channel Surface EMG Recording and Motor Unit Decomposition

For the recording of surface EMG signals and their decomposition into individual motor units, the subjects performed submaximal isometric knee extension. After the MVC recording, the subjects performed ramp contractions from 0 to 90% of MVC in 45 s (rate of force increase: 5% MVC/s). The MVC forces used to set the target force were the values measured in the same period. Target and actual forces were visually presented to the subjects on a monitor. Two or three trials were performed with a >2-min rest interval between them. Out of two or three trials, the one trial with the smaller error between the target and actual forces was selected for analysis. The present study needs to identify motor unit recruitment threshold and to calculate motor unit firing rate at various force levels. While sustained contraction at a constant force level could provide more stable firing rates, large number of contractions would be needed for our purposes. The present study thus chose ramp contraction for analyzing the detailed motor unit firing patterns.

Surface EMG signals from the VL muscle were recorded with a semi-disposable adhesive grid of 64 electrodes (ELSCH064R3S, OT Bioelectronica, Torino, Italy). The electrode grid is made of 13 rows and 5 columns of electrodes with 1 mm diameter and 8 mm inter-electrode distance with one missing electrode at the upper left corner. The method used for determining the electrode location was as previously described (Watanabe et al., 2013, 2016). Briefly, the center of the electrode grid was located at the mid-point of the longitudinal axis of the VL muscle, i.e., the line between the head of the greater trochanter and inferior lateral edge of the patella, and the columns were aligned along the VL longitudinal axis. A reference electrode (C-150, Nihon Kohden, Tokyo, Japan) was placed at the iliac crest. Monopolar surface EMG signals were recorded and amplified by a factor of 500, sampled at 2,048 Hz, and converted to digital form by a 12-bit analog-to-digital converter (EMG-USB, OT Bioelectronica, Torino, Italy) together with the force transducer signal. Recorded monopolar surface EMG signals were transferred to analysis software (MATLAB R2009b, MathWorks GK, Tokyo, Japan), filtered by a band-pass filter (10–450 Hz), and differentiated between neighboring electrodes along the columns. Fifty-nine bipolar surface EMG signals were used for further analysis.

Bipolar surface EMG signals were decomposed with the Convolution Kernel Compensation (CKC) technique into individual motor units (Holobar and Zazula, 2004; Holobar and Zazula, 2008; Merletti et al., 2008; Holobar et al., 2009). As details of the procedures of decomposition analysis have already been reported (Holobar et al., 2009; Farina et al., 2010; Gallego et al., 2015a,b; Yavuz et al., 2015), only a brief outline is provided here. The pulse-to-noise ratio (PNR), introduced by Holobar et al. (2014), was used as a reliable indicator of the motor unit identification accuracy (Holobar et al., 2014). Only motor units with PNR > 30 dB (corresponding to an accuracy of motor unit firing identification > 90%) were used for further analysis, and all other motor units were discarded (Holobar et al., 2014). Identified motor units were manually verified on visual inspection by 1 investigator, and the results were checked by another investigator. After the decomposition, instantaneous firing rates of individually identified motor units were calculated from the discharge times. Inter-discharge intervals <33.3 or >250 ms were excluded from the calculation of the firing rate, since they lead to an unusually high (>30 Hz) or low (<4 Hz) instantaneous firing rate. This range of the unusual firing rate was set according to previous studies using the same muscle (Adam and De Luca, 2005; Welsh et al., 2007; Holobar et al., 2009; Watanabe et al., 2013). Mean values of the instantaneous firing rate during each 10% increment of MVC were calculated for individual motor units of each subject as the mean firing rate. Each 10% increment of MVC was set from the MVC force at 0 week for all three periods. Therefore, for example, mean firing rates at 50% of MVC at 0, 3, and 6 weeks were calculated at the same absolute force. Firing rates were excluded from further analysis when the coefficient of variation in mean firing rate was greater than 30% or when the number of firings was lower than two, i.e., one firing (Fuglevand et al., 1993). We divided the detected motor units into three motor unit groups by recruitment threshold, i.e., motor units recruited at <20%, 20–40%, and 40–60% of MVC (Watanabe et al., 2016). Since this calculation was also based on the MVC at 0 week, recruitment thresholds of each motor unit group were the same absolute forces within a subject at all three periods. These procedures for decomposition analysis and calculation of firing rate of individual motor units were already used in our previous studies (Watanabe et al., 2013, 2016).

### Anthropometric Tests

Body composition was estimated from body impedance measurements (InBody270, InBody Japan Inc., Tokyo, Japan) and the body weight, whole body muscle mass, fat mass, and its percentages and leg muscle mass were used for further analysis. Thicknesses of the VL muscles and subcutaneous skin tissue were measured by ultrasonographic imaging (FAZONE CB, FUJI FILM, Tokyo, Japan). Longitudinal B-mode images were taken at 50% of the distance between the head of the greater trochanter and inferior lateral edge of the patella, at the location of the center of the electrode grid for surface EMG recording (Watanabe et al., 2013, 2016, 2018). The vertical distances between the skin surface and the superficial edge of the VL muscle as subcutaneous tissue and between superficial edge of the VL muscle and superficial edge of the femur as muscle thickness were measured with image analysis software (Image J, National Institutes of Health, Maryland, United States). A single operator blinded to information on the subjects and test periods carried out this analysis.

### Reproducibility

We performed the same test at 3 weeks before the intervention. Using the pre-test data, we calculated the intraclass correlation coefficient (ICC) for each measurement except for the motor unit firing pattern. ICCs between the pre-test and test at 0 week were 0.992 and 0.999 for the muscle mass and body fat measured by the body impedance method, respectively, 0.696 and 0.936 for muscle and subcutaneous tissue thickness by ultrasonography, respectively, 0.983 for MVC, 0.942 for the chair-stand test, and 0.900 for gait speed.

### Statistics

All data are provided as the mean ± SD. Since our results included non-normal distributed data which are tested by the Shapiro–Wilk test, non-parametric statistical tests were used in the present study. The Kruskal–Wallis test was used to detect the effect of group on the number of males, age, height, body mass, body fat and muscle mass estimated by InBody, muscle and subcutaneous tissue thicknesses, MVC, result of the chair-stand test, gait time, total energy and protein intakes at 0 week, and test meal ingestion timing. To test the effect of intervention, the Friedmann test was applied to the body mass, body fat and muscle mass estimated from InBody, muscle and subcutaneous tissue thicknesses, MVC, result of the chair-stand test, and gait time of 0, 3, and 6 weeks for each subject group. If significant effects of intervention were detected, the Bonferroni–Dunn test was used to compare the values among three periods (Pereira et al., 2015). For the motor unit firing rate, the effect of intervention was assessed by the Kruskal–Wallis test for each motor unit group of the four different groups, respectively. When a significant effect of the intervention was identified by the Kruskal–Wallis test, firing rates were compared among 0, 3, and 6 weeks by the Dunn’s test (Katz, 2006). For nutritional parameters, the Kruskal–Wallis test were used to assess the effects of groups at PRE. When a significant effect of the group was identified by the Kruskal–Wallis test, nutritional parameters were compared among four groups by the Dunn’s test (Katz, 2006). Also, nutritional parameters between before and during the intervention were compared by Wilcoxon rank sum test within a group. The level of significance was set at *P* < 0.05. For the *post hoc* tests after the Friedmann test and the Kruskal–Wallis test such as Bonferroni–Dunn test and the Dunn’s test, *p*-values were modified by Bonferroni correction (Katz, 2006; Pereira et al., 2015). The epsilon-squared estimate of effect size (ε^2^) was additionally calculated and this value from 0 to 1 indicates no relationship to a perfect relationship (Tomczak and Tomczak, 2014). Statistical analysis was performed using SPSS (version 15.0, SPSS, Tokyo, Japan) and MATLAB (R2009b, MathWorks GK, Tokyo, Japan).

## Results

At the beginning of intervention (0 week), there were no significant differences among the groups in the number of males (*p* = 0.995), age (*p* = 0.643), height (*p* = 0.023), body mass (*p* = 0.442), body fat (*p* = 0.431) and muscle mass estimated by InBody (*p* = 0.673), muscle (*p* = 0.512) and subcutaneous tissue thicknesses (*p* = 0.490), MVC (*p* = 0.966), result of the chair-stand test (*p* = 0.455), or gait time (*p* = 0.516) (Table 1). No significant effect of group on test meal ingestion timing (*p* < 0.05) (Table 2).

**Table 2:** Results of diet survey using a food frequently questionnaire (FFQg) and test meal ingestion timing.

		APP-CN		PLA-CN		APP-RT		PLA-RT	
Total energy	PRE	1835 ± 407		1805 ± 394		2154 ± 659		1923 ± 189	
(kcal)	3–6 weeks	1976 ± 407	*p* < 0.001	1982 ± 296	*p* < 0.026	2296 ± 659	*p* < 0.001	2121 ± 303	*p* < 0.016
Protein	PRE	67.7 ± 14.6		66.7 ± 19.8		74.0 ± 23.5		68.5 ± 9.9	
(g)	3–6 weeks	78.2 ± 15.4	^∗^*p* = 0.004	73.4 ± 18.9	^∗^*p* =0.021	83.6 ± 25.6	^∗^*p* = 0.033	78.0 ± 10.9	^∗^*p* = 0.023
Total energy	PRE	35.2 ± 10.9		31.0 ± 7.2		40.1 ± 13.0		34.3 ± 7.0	
(kcal/kg)	3–6 weeks	38.0 ± 11.2	*p* = 0.001	34.3 ± 7.0	^∗^*p* = 0.033	42.4 ± 13.0	^∗^*p* = 0.033	37.6 ± 8.4	*p* = 0.019
Protein	PRE	1.3 ± 0.4		1.1 ± 0.3		1.4 ± 0.6		1.2 ± 0.3	
(g/kg)	3–6 weeks	1.5 ± 0.4	*p* = 0.004	1.3 ± 0.4	*p* = 0.021	1.6 ± 0.7		1.4 ± 0.3	*p* = 0.023
Fat	PRE	1.3 ± 0.5		1.1 ± 0.3		1.4 ± 0.6		1.2 ± 0.3	
(g/kg)	3–6 weeks	1.4 ± 0.5		1.2 ± 0.4		1.5 ± 0.7		1.3 ± 0.4	
Carbohydrate	PRE	4.3 ± 1.4		3.7 ± 1.2		5.1 ± 1.7		4.1 ± 1.1	
(g/kg)	3–6 weeks	4.7 ± 1.2	*p* = 0.007	4.2 ± 0.9	*p* = 0.021	5.3 ± 1.9		4.5 ± 1.1	
Protein (g/kg) (3–6 weeks/PRE, %)r		117.8 ± 20.5		111.9 ± 12.2		115.5 ± 20.1		115.5 ± 19.2	
Ingestion timing	Breakfast	44.7 ± 32.0		38.1 ± 36.6		48.4 ± 44.0		34.3 ± 33.0	
(%)	Lunch	28.4 ± 22.5		27.2 ± 31.8		24.3 ± 28.9		23.0 ± 27.3	
	Dinner	26.9 ± 23.1		34.7 ± 32.3		27.2 ± 27.8		42.7 ± 35.7	
							*p* < 0.05 ^∗^ vs. PRE		


*^∗^p < 0.05 vs. PRE.*

Total energy, protein, and total energy normalized by body weight at 3–6 weeks were significantly greater than those at PRE in all groups (*p* < 0.05) (Table 2). Protein normalized by body weight significantly increased from PRE to 3–6 weeks for APP-CN (*p* = 0.004), PLA-CN (*p* = 0.021), and PLA-RT (*p* = 0.023), but not for APP-RT (*p* = 0.062) (Table 2). Carbohydrate normalized by body weight significantly increased from PRE to 3–6 weeks for APP-CN (*p* = 0.007) and PLA-CN (*p* = 0.021) (Table 2).

No significant effects of intervention were noted on the body mass, body fat and muscle mass estimated by InBody, muscle and subcutaneous tissue thicknesses, MVC, or gait time in any of the groups (*p* < 0.05) (Table 3). For the chair-stand test, significant increases were observed at 6 weeks compared with 0 week in all groups (*p* < 0.05) (Table 3). Significant increases from 0 to 3 weeks were observed in APP-RT (*p* = 0.012), but not in other groups (*p* > 0.05) (Table 3).

**Table 3:** Results of anthropometric parameters and motor function tests.

		APP-CN		PLA-CN		APP-RT		PLA-RT	
Body mass (kg)	0 week	53.9 ± 8.9		60.3 ± 10.8		56.2 ± 10.8		57.1 ± 8.6	
	3 weeks	53.9 ± 9.0		60.4 ± 10.9		56.7 ± 11.1		57.8 ± 8.3	
	6 weeks	53.7 ± 8.7		59.8 ± 10.4		57.0 ± 10.7		57.7 ± 8.1	
Body fat (%)	0 week	26.0 ± 6.6		27.7 ± 5.8		23.4 ± 5.9		25.3 ± 6.0	
	3 weeks	25.9 ± 6.6		28.2 ± 5.7		24.1 ± 5.7		26.1 ± 6.1	
	6 weeks	25.6 ± 6.3		27.6 ± 6.1		23.9 ± 5.9		25.4 ± 6.6	
Muscle mass (kg)	0 week	21.5 ± 4.2		23.5 ± 4.7		23.5 ± 5.2		23.3 ± 4.4	
	3 weeks	21.6 ± 4.3		23.4 ± 4.5		23.4 ± 5.3		23.3 ± 4.5	
	6 weeks	21.6 ± 4.3		23.4 ± 4.5		23.7 ± 5.3		23.6 ± 4.6	
Muscle thickness (cm)	0 week	3.52 ± 0.63		3.94 ± 0.99		3.84 ± 0.79		3.94 ± 0 52	
	3 weeks	3.40 ± 0.69		3.89 ± 0.97		3.87 ± 0.87		3.85 ± 0.57	
	6 weeks	3.56 ± 0.69		3.93 ± 0.89		3.81 ± 0.84		3.91 ± 0.49	
ST thickness (cm)	0 week	0.68 ± 0.30		0.79 ± 0.28		0.78 ± 0.31		0.68 ± 0.25	
	3 weeks	0.74 ± 0.33		0.81 ± 0.23		0.81 ± 0.33		0.66 ± 0.28	
	6 weeks	0.71 ± 0.26		0.78 ± 0.23		0.80 ± 0.32		0.75 ± 0.30	
MVC (Nm)	0 week	340.0 ± 111.3		350.0 ± 87.8		374.6 ± 131.3		356.4 ± 118.1	
	3 weeks	339.6 ± 111.2		351.3 ± 89.0		383.2 ± 129.0		379.2 ± 111.0	
	6 weeks	340.8 ± 96.6		361.7 ± 101.4		387.2 ± 128.3		379.8 ± 106.7	
Chair stand (times)	0 week	16.9 ± 3.6		17.1 ± 3.7		18.2 ± 1.9		18.2 ± 3.3	
	3 weeks	17.5 ± 4.2		17.8 ± 3.6		19.8 ± 2.2	^∗^*p* = 0.012	18.9 ± 3.8	
	6 weeks	18.5 ± 4.2	^∗^*p* = 0.015	18.4 ± 3.5	^∗^*p* = 0.03	21.2 ± 1.9	^∗#^*p* = 0.006 vs. 0 week	20.5 ± 3.2	^∗#^*p* = 0.006 vs. 0 week
Gait time (sec.)	0 week	7.1 ± 0.8		7.5 ± 1.1		7.2 ± 0.9		6.9 ± 1.0	
	3 weeks	7.3 ± 1.1		7.6 ± 1.0		7.2 ± 1.2	*p* = 0.018 vs. 3 weeks	6.9 ± 1.2	*p* = 0.012 vs. 3 weeks
	6 weeks	7.0 ± 1.0		7.4 ± 1.1		7.1 ± 1.5		6.7 ± 1.1	
Leg press 1RM (kg)	0 week					106.3 ± 40.8		106.3 ± 34.0	
	3 weeks					120.5 ± 42.5		124.4 ± 38.2	
	6 weeks					137.4 ± 45.0		137.5 ± 39.7	
								^∗^ vs. 0 week, ^#^ vs. 3 weeks	


*^∗^p < 0.05 vs 0 week, ^#^*p* < 0.05 vs 3 week.*

**FIGURE 1:**
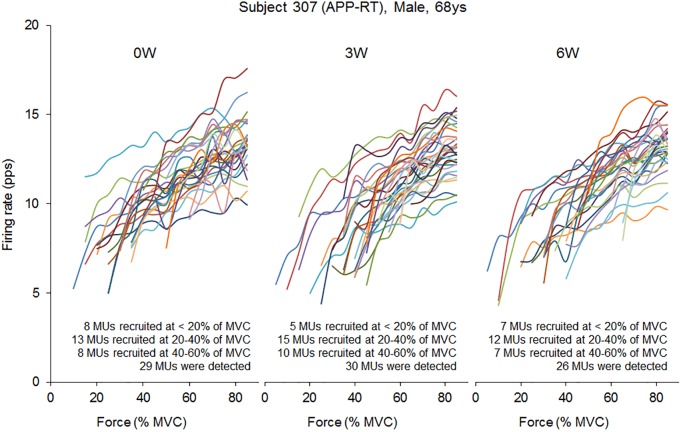
Firing rate of individual motor units in a representative subject from APP-RT during ramp contraction at 0, 3, and 6 weeks.

**FIGURE 2:**
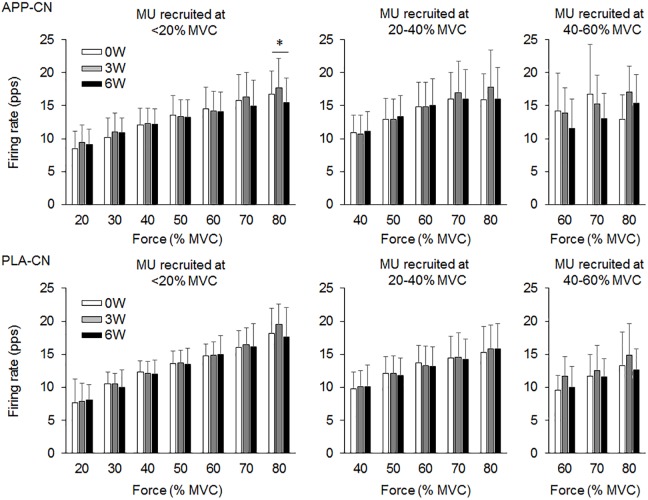
Motor unit firing rates of the group with fish protein intake (APP-CN) and the group with the placebo (PLA-CN). MU, motor units. The symbol ^∗^ indicates significant differences (*p* < 0.05) among the periods.

**FIGURE 3:**
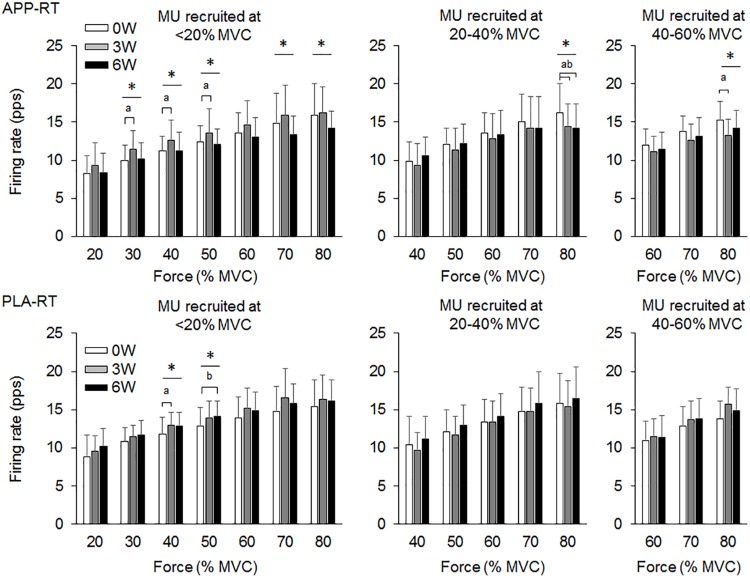
Motor unit firing rates of the group with fish protein intake and resistance training (APP-RT) and the group with placebo and resistance training (PLA-RT). MU, motor units. The symbols ^∗^, ^a^, and ^b^ indicate significant differences (*p* < 0.05) among the periods, between 0 and 3 weeks, and between 0 and 6 weeks, respectively.

In the present study, total 1,204 motor units were detected from 50 subjects at three periods and considered for analysis (Table 4). Representative data from a subject of APP-RT was shown in Figure 1. In PLA-CL, none of the firing rates of any motor unit groups significantly changed during intervention (*p* > 0.05) (Figure 2). In APP-CN, a significant effect of intervention was noted in the firing rate of motor units recruited at <20% of MVC at 80% of MVC (*p* = 0.045, ε^2^ = 0.06) (Figure 2). In PLA-RT, a significant effect of intervention was noted in the firing rate of motor units recruited at <20% of MVC at 40 (*p* = 0.030, ε^2^ = 0.08) and 50% of MVC (*p* = 0.028, ε^2^ = 0.07) (Figure 3). Firing rates of motor units recruited at <20% of MVC at 3 weeks at 40% of MVC (*p* = 0.044) and at 6 weeks at 50% of MVC were significantly higher than that at 0 week in PLA-RT (*p* = 0.031) (Figure 3). In APP-RT, a significant effect of intervention was observed in the firing rate of motor units recruited at <20% of MVC at 30 (*p* = 0.011, ε^2^ = 0.09), 40 (*p* = 0.004, ε^2^ = 0.10), 50 (*p* = 0.017, ε^2^ = 0.08), and 70% of MVC (*p* = 0.003, ε^2^ = 0.11), in the firing rate of motor units recruited at 20–40% of MVC at 80% of MVC (*p* = 0.005, ε^2^ = 0.08), and in firing rate of motor units recruited at 40–60% of MVC at 80% of MVC (*p* = 0.001, ε^2^ = 0.18) (Figure 3). For motor units recruited at <20% of MVC, firing rates at 3 weeks were significantly higher than those at 0 week at 30 (*p* = 0.011), 40 (*p* = 0.004), and 50% of MVC in APP-RT (*p* = 0.032) (Figure 3). Firing rates of motor units recruited at 20–40 of MVC at 0 week were significantly higher than those at 3 weeks (*p* = 0.012) and 6 weeks (*p* = 0.018) at 80% of MVC in APP-RT (Figure 3). Firing rates of motor units recruited at 40–60 of MVC at 0 week were significantly higher than those at 3 weeks (*p* = 0.001) at 80% of MVC in APP-RT (Figure 3).

**Table 4:** Number of motor units detected and considered for analysis for each group.

		Number of detected motor units at each force level (% MVC)								
**APP-CN (312 MUs)**									
Recruitment threshold		20	30	40	50	60	70		80
<20% MVC	0 week	36	36	35	36	36	36		33
	3 weeks	40	41	41	41	41	27		23
	6 weeks	49	50	50	50	50	50		47
20–40% MVC	0 week			42	44	44	43		37
	3 weeks			48	50	50	46		34
	6 weeks			64	52	52	52		42
40–60% MVC	0 week					11	12		7
	3 weeks					7	7		7
	6 weeks					8	8		8
**PLA-CN (243 MUs)**									
Recruitment threshold		20	30	40	50	60	70		80
<20% MVC	0 week	18	18	18	18	18	18		18
	3 weeks	21	21	21	21	21	21		11
	6 weeks	17	19	19	19	19	19		16
20–40% MVC	0 week			49	49	49	49		46
	3 weeks			43	43	43	43		26
	6 weeks			41	41	42	41		36
40–60% MVC	0 week					21	21		21
	3 weeks					19	19		17
	6 weeks					11	11		11
**APP-RT (330 MUs)**									
Recruitment threshold		20	30	40	50	60	70		80
<20% MVC	0 week	42	42	42	42	42	42		42
	3 weeks	34	34	34	34	34	34		32
	6 weeks	29	29	29	29	29	29		27
20–40% MVC	0 week			43	44	44	44		44
	3 weeks			57	59	59	59		55
40–60% MVC	6 weeks			34	34	34	34		31
	0 week					36	36		36
	3 weeks					25	25		25
	6 weeks					27	27		26
**PLA-RT(319MUs)**									
Recruitment threshold		20	30	40	50	60	70		80
<20% MVC	0 week	42	42	42	42	42	40		34
	3 weeks	44	44	44	44	44	44		43
	6 weeks	27	27	27	27	27	27		25
20–40% MVC	0 week			46	47	47	45		36
	3 weeks			55	56	56	56		52
	6 weeks			45	46	46	46		40
40–60% MVC	0 week					10	10		10
	3 weeks					22	23		19
	6 weeks					24	24		24


## Discussion

We investigated the effect of fish protein intake and resistance training on the motor unit firing pattern and motor function in the elderly. No significant differences in dietary protein intake during the intervention were noted among the four groups (Table 3). While the parameters associated with the muscle mass, MVC, and normal gait speed were not significantly changed during the intervention in any group (*p* > 0.05), the chair-stand test results were significantly improved following 6 weeks of intervention in APP-CN, PLA-CN, and PLA-RT (*p* < 0.05) and at 3 and 6 weeks of intervention in APP-RT (*p* < 0.05) (Table 2). Significant increases in firing rates of motor units with low-recruitment thresholds were observed in PLA-RT at 3 and 6 weeks (*p* < 0.05) (Figure 3). In APP-RT, significant increase in firing rates of motor units with low-recruitment thresholds were noted at 3 weeks (*p* < 0.05), but not at 6 weeks (*p* > 0.05) (Figure 3), meaning that the time course of the firing rate adaptations in motor units with a low-recruitment threshold was not consistent with PLA-RT (Figure 3). Also, APP-RT showed significant decreases in firing rate of motor units with middle-recruitment thresholds (*p* < 0.05) (Figure 3). These findings support the hypothesis that resistance training increases the firing rates of motor units with low-recruitment thresholds. The hypothesis that fish protein intake induces an increase in the muscle volume was not supported.

### Effect of Exercise and Nutritional Interventions on Motor Functions

The chair-stand test results were improved during 6 weeks of intervention in all groups, including those with no exercise intervention, i.e., APP-CN and PLA-CN (Table 2). For the chair-stand test in the elderly, while a high test–retest intra-class correlation was confirmed (Jones et al., 1999), a learning effect of repeated measurements was also reported (Tager et al., 1998). The subjects in this study performed four trials of the chair-stand test to familiarize themselves with it before each measurement. This may have introduced a learning effect that consequently improved the test performance in all the subject groups. For the groups with resistance training intervention, i.e., APP-RT and PLA-RT, significant differences were also observed between 3 and 6 weeks (*p* < 0.05) (Table 2). This means that the intervention of resistance training leads to a more marked improvement in the chair-stand test results. The leg-press exercise employed for resistance training in the present study and standing up from and sitting on a chair are similar lower extremity movements. Also, a high positive correlation between the results of the chair-stand test and maximum leg-press performance was confirmed (Jones et al., 1999). Therefore, it is reasonable to consider that resistance training involving leg-press exercise may improve the results of the chair-stand test.

For APP-RT, a significant improvement in the chair-stand test performance was noted between 0 and 3 weeks in addition to significant changes between 0 and 6 weeks observed in all groups and between 3 and 6 weeks observed in the groups with resistance training intervention (Table 2). From this result, we suggest that the intake of fish protein during resistance training facilitates earlier improvement of the motor function. Although it is difficult to discuss the physiological mechanisms behind this characteristic adaptation in APP-RT accurately in this study, we provide further discussion on the effect of resistance training with nutritional intervention on the motor unit firing pattern.

### Effect of Resistance Training on Motor Unit Firing Pattern

Leong et al. (1999) demonstrated that the maximal motor unit firing rate of the knee extensor muscle in well-trained older weight lifters with a minimum of 5 years of training experience was significantly higher than that in an age-matched control group (Leong et al., 1999). This means that chronic resistance training can improve the motor unit firing pattern even in the elderly. In PLA-RT, the firing rate of motor units recruited at <20% of MVC significantly increased at 3 and 6 weeks compared with 0 week at 40 and 50% of MVC (*p* < 0.05) (Figure 3). Increases in the motor unit firing rate were also noted in APP-RT, but not in APP-CN or PLA-CN (Figure 2). These results suggest that the firing rate of motor units increases following resistance training in the elderly. In young populations, Vila-Cha et al. (2010) reported an increase in the motor unit firing rate at 30% of MVC following 6 weeks of resistance training in the vasti muscles (Vila-Cha et al., 2010). This supports our results. On the other hand, Kamen and Knight (2004) showed that a 6-week resistance training did not lead to a significant increase in the motor unit firing rate during maximal and submaximal contractions in the elderly (Kamen and Knight, 2004). On the other hand, Pucci et al. (2006) reported a decrease in the motor unit firing rate at the same absolute force level during 3 weeks of isometric training in the quadriceps femoris muscles (Pucci et al., 2006). Other studies demonstrated no significant changes in the motor unit firing rate during submaximal contraction in the vastus lateralis muscles (Rich and Cafarelli, 2000; Kamen and Knight, 2004). These variations in the acute effect of resistance training on the motor unit firing rate would be primarily due to differences in the contraction type, intensity, duration, volume, and/or frequency of resistance training. Also, we should note that while these previous studies showed an increase in MVC following training, our study revealed an improvement in the 1RM on leg-press exercise, but not in MVC.

While the effect of chronic and/or acute resistance training on the motor unit firing rate has been investigated in several studies, the recruitment threshold of individual motor units has not been considered. This is the first study to separate the detected motor units into those with different recruitment thresholds in order to calculate the firing rate during training intervention. In the present study, the effect of resistance training on the firing rate was not uniform among the motor unit groups recruited at different force levels (Figure 3). Increases in the firing rate during resistance training were mainly noted in motor units recruited at <20% of MVC for APP-RT and PLA-RT, and these increases were noted at low to moderate force levels, i.e., 30–50% of MVC, but not at higher force levels (Figure 3). These findings suggest that alterations in the motor unit firing rate following resistance training in the elderly occurred in a limited number of motor unit groups and force ranges. Since we applied 70% of 1RM in the resistance training and the training intensity was revised in the middle of training, a large range of motor units including high-recruitment threshold motor units would be recruited during the resistance training. In fact, it was demonstrated that muscle fiber hypertrophy occurs in both fast and slow twitch fibers during resistance training even in the elderly (Brown et al., 1990). We therefore suggest that although all or most motor units are recruited during training, adaptations in rate coding were not uniform among the recruited motor units (Figure 3). In our previous study, differences in firing rates between the elderly and young were mainly noted in motor units recruited at low force levels (Watanabe et al., 2016). From the results of the present and previous studies, we suggest that the response of low-recruitment threshold motor units might be more plastic to the effect of resistance training and aging.

### Effect of Resistance Training With Nutritional Intervention on Motor Unit Firing Pattern

While similar results, i.e., no significant changes in the motor unit firing rate, were observed between APP-CN and PLA-CN (Figure 2), different patterns of adaptations in the motor unit firing rate were noted between APP-RT and PLA-RT (Figure 3). These findings indicate that the motor unit firing pattern is not affected by only APP intake, but could be modified by APP intake in conjunction with resistance training. It is well-known that resistance training improves the muscle strength and stimulates muscle protein synthesis even in the elderly (Dickinson et al., 2013) and that protein intake has a beneficial effect (Houston et al., 2008). In this study, although additional protein was given to APP-CN and APP-RT during the intervention, protein intake volumes were controlled among the groups with and without fish protein (Table 3). We thus consider that the results in APP-RT regarding motor performance and motor unit firing patterns would be specific to APP, and not to the protein intake volume. Since it would be unlikely for APP to act directly on the central nervous system, we suggest that APP acts on the motor unit firing pattern via the processes of muscle protein metabolism. In a previous study, the gastrocnemius muscle weight in rats was increased with APP intake, but not with casein intake, in 6 or 8 weeks with the control of protein volumes on comparing APP and casein groups (Kawabata et al., 2015). They also noted a significant increase of myosin heavy chain gene expression in *Myh4*, which is related to fast-twitch muscle fibers in the APP intake group. Although these findings were observed in rat muscles, it is considered that muscle protein metabolism can also be modified by APP intake in the human body. However, the present study didn’t find detectable changes in muscle volume in ultrasonography and body impedance method in all groups including the groups with APP intake (Table 2). This would be caused by some reasons, i.e., 6 weeks intervention was short, training frequency (two times per week) was low, or measured muscle was limited to one of the four components of trained muscle group. On the other hand, training stimulation in the present study would be sufficient to have an effect on muscle protein metabolism. Muscle proteins are constantly being broken down and synthesized, and they are controlled predominantly by physical activity and food consumption (Landi et al., 2016; Tipton et al., 2018). Therefore, at the muscle cell level, processes of muscle protein metabolism could be influenced by APP, even in a short period such as 6 weeks. While detailed signaling from peripheral muscle to the central nervous system is difficult to determine in this study, a modification of muscle protein processes by APP intake may play a role in the increase and/or decrease of the motor unit firing rates.

In motor units recruited at <20% of MVC, significant increases in the firing rate occurred at 3 and 6 weeks in PLA-RT, but at only 3 weeks in APP-RT (*p* < 0.05) (Figure 3). These results suggest that the time course of adaptations in the motor unit firing rate during resistance training is modified by APP intake. Few previous studies showed time course changes in the motor unit firing rate following resistance training intervention. Patten et al. (2001) and Kamen and Knight (2004) showed decreased and unchanged maximal motor unit firing rates following 6 weeks of the resistance training intervention after a rapid increase in the motor unit firing rate within 2 or 7 days after a baseline test (Patten et al., 2001; Kamen and Knight, 2004). Vila-Cha et al. (2010) reported an increase in the motor unit firing rate at 30% of MVC following resistance training at 3 and 6 weeks when compared with 0 week, but an increment was not observed from 3 to 6 weeks (Vila-Cha et al., 2010). In this previous study, the motor unit firing rate of vastus medialis muscle at 10% of MVC increased at 3 weeks and returned toward the baseline at 6 weeks. From the results of previous studies (Patten et al., 2001; Kamen and Knight, 2004; Vila-Cha et al., 2010), the motor unit firing rate following resistance training could be increased in the early phase and be maintained or decreased after this phase. Therefore, reduction in the motor unit firing rate following resistance training could be explained by matching the motor unit firing rate with muscle contractile properties, such as a decrease in the proportion of type IIB fibers and increase in that of type IIA fibers following resistance training (Staron et al., 1990; Staron et al., 1994). Also, reduction in the motor unit firing rate at the same absolute force level after resistance training reveals that the lower motor unit firing rate can generate the same absolute force following resistance training. Kamen and Knight (2004) concluded that the motor unit firing rate adapts to the modification of muscle contractile properties based on the training stimulus after a rapid increase in the motor unit firing rate (Kamen and Knight, 2004). In the present study, motor unit firing rate reduction was observed in APP-RT, but not in PLA-RT. We suggest that APP intake enhanced some process of muscle protein synthesis and then accelerated the time course of adaptation in the motor unit firing pattern.

While changes in the motor unit firing rate were noted only in motor units with a low-recruitment threshold for PLA-RT, significant changes were noted in motor units with both low- and high- recruitment thresholds for APP-RT (Figure 3). Only animal studies had divided motor units by their recruitment threshold to investigate the effect of exercise intervention on the motor unit firing rate. In rat muscles, daily spontaneous running such as endurance training induced adaptations of electrophysiological properties in slow motoneurons but not in fast motoneurons (Beaumont and Gardiner, 2002). This suggests that training-specific adaptations could occur in slow motoneurons. In the present study, resistance training with the same intensity and volume was applied to APP-RT and PLA-RT. Under the same training stimulus conditions, types of recruited motor units and muscle fibers should have been similar among subjects in APP-RT and PLA-RT. Therefore, decrease in firing rates of motor units with a high-recruitment threshold in APP-RT could not be due to differences in the training regimen. On the other hand, alterations in contractile properties of muscle fibers may also induce modifications in the motor unit firing rate. Relationships between muscle fiber contractile properties and the motor unit firing pattern have been reported (Herda et al., 2015, 2016; Trevino and Herda, 2016; Trevino et al., 2016). As described above, APP intake would not act directly on the central nervous system. We thus suggest that APP intake with resistance training modifies the contractile properties of muscle fibers that are innervated by motor neurons with a high-recruitment threshold in addition to the contractile properties of muscle fibers that are innervated by motor neurons with a low-recruitment threshold. These alterations in the contractile properties of different types of muscle fibers could lead to changes in the firing patterns in motor units with low- and high-recruitment thresholds. However, we should note that further studies are needed to clarify the mechanisms of adaptations in the motor unit firing rate following resistance training and nutritional supplementation.

### Limitations

In the present study, motor unit firing rates were compared among three different periods: 0, 3, and 6 weeks. Although we endeavored to place electrodes at the same position each time using bone markers as a reference, there was no supporting evidence that action potentials from the same motor units were recorded or that the same motor units were detected by decomposition analysis. On the other hand, there were no significant changes in the thickness of the VL muscle or subcutaneous tissues under the electrodes during the intervention (*p* > 0.05) (Table 3), suggesting that marked changes in anatomical properties did not occur in the investigated area. We also should note possibility of changes in motor unit recruitment thresholds following the interventions. Since the present study didn’t track same motor units among three periods during the intervention, same motor units may be categorized as different motor unit group among the periods. However, change in motor unit recruitment threshold could not be greater than 20% of MVC which used as force levels to categorize motor unit groups. We thus assumed the detected motor unit populations within the motor unit groups did not critically vary during the different periods in this study.

The present study detected large number of motor units in total (1,204). However, numbers of detected motor units were different depending on recruitment thresholds and those for 40–60% MVC of recruitment threshold were smaller than those for <20% and 20–40% MVC of recruitment threshold (Table 4). Therefore, firing rate of motor units with different recruitment thresholds were calculated from different numbers of samples. Also, motor unit firing rates from all subjects were not included in the calculated motor unit firing rates at each period and motor unit group. In the present study, motor units recruited at <20% MVC, 20–40% MVC, and 40–60% MVC were detected from 12, 11, and 9 of 13 subjects in APP-CN, 8, 11, and 7 of 12 subjects in PLA-CN, 10, 11, and 9 of 12 subjects in APP-RT, 11, 12, and 11 of 13 subjects in PLA-RT. Variations in number of detected motor units among the subjects, groups, and recruitment thresholds may induce difference in the statistical powers among the subject groups and/or motor unit groups.

This study investigated right leg only for MVC, muscle thickness, and surface EMG, while dominant leg was not identified for each subject. For APP-RT and PLA-RT, the subjects performed bilateral horizontal leg press exercise as resistance training. This exercise may lead leg-dependent training effects since the foot plate of our training system was not separated for each leg and it is difficult to estimate balance of force productions by right or left legs. We should note that this is one of the limitations of this study.

## Conclusion

We investigated the effect of resistance training and fish protein intake for 6 weeks on the motor unit firing pattern and motor function in elderly. Improvement of motor function and changes in motor unit firing pattern were observed in the groups with the resistance training, but not in the groups without the resistance training. Increases in motor unit firing patterns following the resistance training were mainly observed in motor units with a low-recruitment threshold. The group with the combination of fish protein intake and resistance training showed a greater improvement in the motor function, a different time course of alterations in firing rates of motor units with a low-recruitment threshold, and decreases in firing patterns of motor units with a high-recruitment threshold. These findings suggest that, in the elderly, the effect of resistance training on the motor unit firing rate can be observed in motor units with a low-recruitment threshold and additional fish protein intake alters the adaptations of motor unit firing patterns and the motor function following resistance training.

## Author Contributions

KW, AH, YM, MK, MO, HA, and TM research concept and design and reviewed the paper. KW, YM, and MO data collection. KW, YM, and AH data analysis. KW wrote the paper.

## Conflict of Interest Statement

The authors declare that the research was conducted in the absence of any commercial or financial relationships that could be construed as a potential conflict of interest.

## Abbreviations

APP: alaska pollack protein
EMG: electromyography
MVC: maximal voluntary contraction
PNR: pulse noise ratio
RM: repetition maximum
